# Stimulus dependence of interocular suppression

**DOI:** 10.1038/s41598-021-88701-x

**Published:** 2021-04-29

**Authors:** Wei Hau Lew, Scott B. Stevenson, Daniel R. Coates

**Affiliations:** University of Houston College of Optometry, 4901 Calhoun Rd, Houston, TX 77004 USA

**Keywords:** Visual system, Perception

## Abstract

Interocular suppression is the phenomenon in which the signal from one eye inhibits the other eye in the presence of dissimilar images. Various clinical and laboratory-based tests have been used to assess suppression, which vary in color, contrast, and stimulus size. These stimulus variations may yield different spatial extents of suppression, which makes it difficult to compare the outcomes. To evaluate the role of stimulus characteristics, we measured the suppression zone using a binocular rivalry paradigm in normally-sighted observers by systematically varying the stimulus parameters. The stimuli consist of a constantly visible horizontal reference seen by one eye while two vertical suppressors were presented to the other eye. With a keypress, the suppressors appeared for 1 s, to induce a transient suppression zone in the middle part of the reference. Subjects adjusted the width between the suppressors to determine the zone. The zone decreased significantly with increasing spatial frequency and lower contrast. The width was 1.4 times larger than the height. The zone was smaller with negative compared to positive contrast polarity but independent of eye dominance, luminance, and colored filters. A departure from scale invariance was captured with a model suggesting a stimulus-dependent and a small fixed non-stimulus-dependent portion.

## Introduction

Binocular rivalry is a visual phenomenon where one experiences the alternating perception of dissimilar images shown to the two eyes. Dissimilar images can result from interocular differences in luminance, contrast, motion, form, colors, and orientation, for example. Dissimilar images do not allow fusion and lead to visual confusion. In order to remove this confusion, each eye will alternately suppress the other eye to exert dominance during binocular rivalry^1^. However, under normal viewing conditions, where the two images are usually similar, observers will fuse the images into a single, unitary percept. Following Wheatstone’s invention of the stereoscope in 1836, researchers have generated a wide variety of artificial situations to explore how binocular vision responds to dramatically different monocular stimuli during rivalry. A common finding is that one will often experience a relatively slow fluctuating “piecemeal” percept consisting of the intermingled monocular images^2^. In the “piecemeal” percept, at a given time, a small portion from one monocular image will dominate and the other percept will be suppressed. Suppression is a defense mechanism needed to resolve the conflicting percepts, to avoid diplopia from mismatched interocular features, and also to facilitate the visibility of the clearer image^3–5^.

While this phenomenon is observed in individuals with normal binocular vision, interocular suppression is also associated with observers with abnormal binocular vision, such as amblyopia or strabismus that arises during development in response to physiological imbalance such as anisometropic optical blur, contrast loss, or misalignment of the eyes^6^. The assessment of suppression plays a crucial role in diagnosis, treatment, and prognosis for amblyopia and strabismus in the clinic. In clinical settings, suppression is assessed with the Worth-4-Dot test (W4D), Bagolini lens, Sbisa Bar and synoptophore. It is challenging for clinicians to compare the result obtained from one test to the other given that each test is designed with different stimulus parameters and dichoptic presentation^7,8^. For example, the W4D test, which uses anaglyph colors, is performed in two different lighting conditions: normal room illumination and in the dark. The two different colors used may introduce some form of binocular dissociation^9^. On the other hand, the Bagolini test uses striated lenses placed in front of the eyes and an observer will perceive orthogonal high spatial frequency stimuli (thin streak of lights). To assess the depth of suppression, the luminance in the fellow eye is decreased by using the Sbisa bar or Neutral Density filters. Meanwhile, the synoptophore testing slides are designed with various object sizes, line thicknesses, colors, and features.

The first study to map the spatial extent of suppression was carried out by Von Graefe in 1896 and followed by Travers using a haploscope system^10^. Over the decades, different sets of stimuli used in laboratory-based tests have shown significant variability^11–17^. In some studies, subjects were confirmed to have suppression with clinical tests during recruitment but had none or minimal suppression with laboratory based-tests^18–20^. Why do these laboratory-based suppression tests fail to replicate the result from clinical tests? One possibility is that the stimulus parameters or presentation mode will affect the spatial extent of suppression. A study by Liu & Schor (1994) found that the size of suppression during binocular rivalry changes with the contrast and spatial frequency of the stimuli^21^. Apart from the spatial characteristics, the dominant eye also suppresses the non-dominant eye longer during the occurrence of binocular rivalry^22,23^. Therefore, temporal factors could also affect the results. Unlike in the clinic, researchers often use brief presentation times and limited response durations.

This study is concerned with the various non-standardized stimulus parameters used in the clinic and in laboratory-based studies. These factors have not been systematically explored in a single study. We posit that these variabilities may affect the nature of the suppression scotoma, causing a change in its size according to the stimulus used. Therefore, here we investigate how stimulus parameters affect the suppression zone in a comprehensive series of experiments by instigating suppression in observers with normal binocular vision, using orthogonal Difference of Gaussian bars in different conditions (spatial frequency, contrast, contrast polarity, eye dominance, luminance, and colored filters). We confirmed that the suppression zone is not fixed, but varies with some (but not all) of these stimuli parameters. While the size of the zone is largely dependent on the spatial frequency of the stimulus, there was a lack of perfect scale invariance, which we captured with a simple model that contained two parts: a stimulus dependent factor and a fixed non-stimulus-dependent factor.

**Figure 1:**
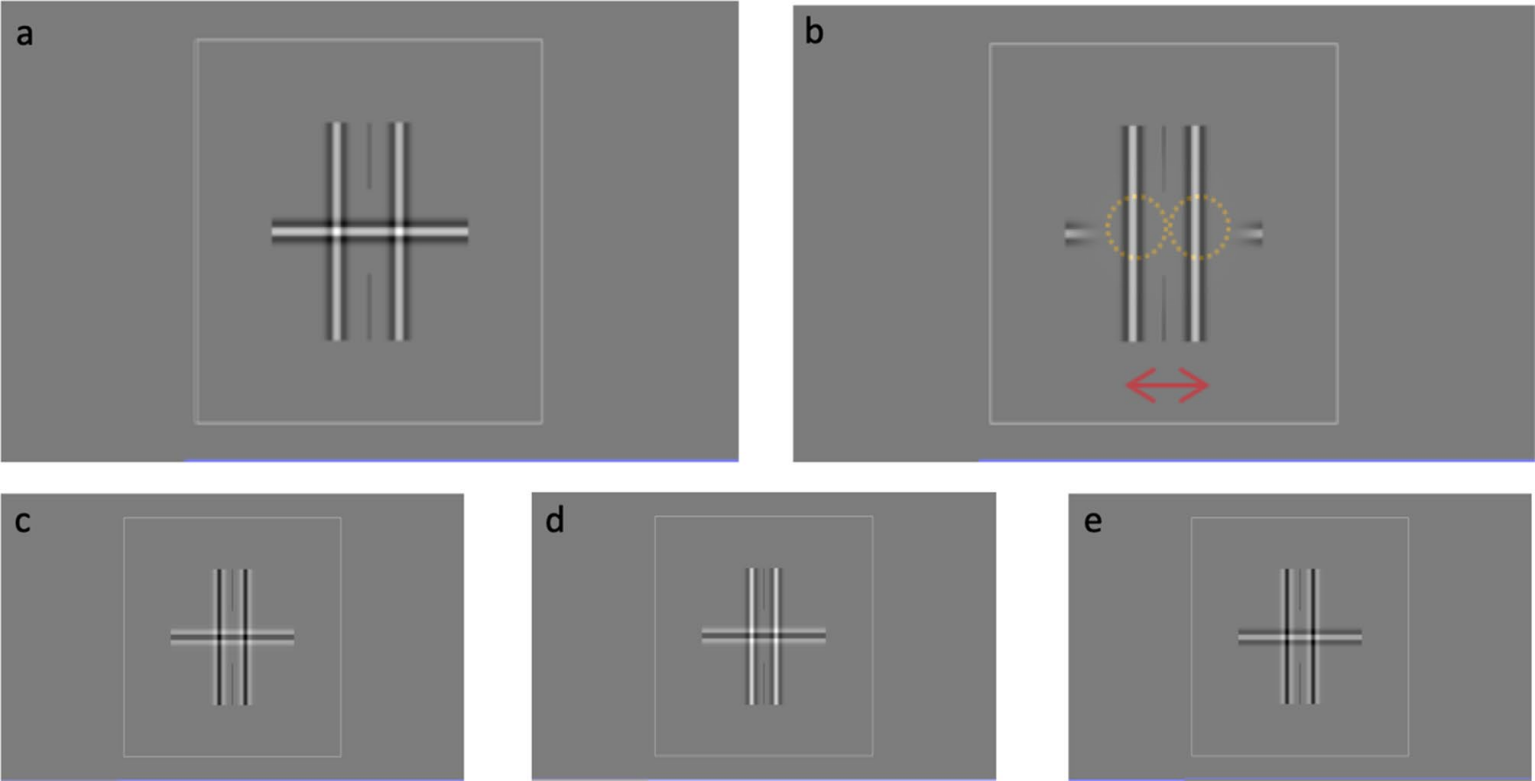
Stimuli used in the experiment. (**a**) The DoG bars had a white center surrounded by a darker grey region on both sides. A white square outline served as the binocular fusion lock. Two fine vertical black lines pointing towards the center part of the reference served as a fixation guide. The horizontal reference bar was presented continuously to one eye. The two vertical suppressors were presented briefly (1 s) to the other eye following a keypress. (**b**) Subjects adjusted the separation of the vertical bars to the widest setting that still suppressed the horizontal segment between them, and could present the suppressors as many times as needed. The distance between the center of the two suppressors is termed as the suppression zone width, (red arrow). An illustration of the area of perceived suppression upon vertical suppressor appearance is outlined by the yellow circles. (**c**–**e**) These figures illustrate the different combinations of contrast polarity tested in Experiment 2B: (**c**) black–black, (**d**) black–white, and (**e**) white–black. The first color in the combination refers to the center of the horizontal reference followed by the vertical suppressors.

To measure the spread of suppression, we adopted the dichoptic stimuli from Liu & Schor (1994), comprising a constantly visible horizontal DoG (reference) seen by one eye with two vertical DoGs (suppressors) that are intermittently presented to the other eye (Fig. 1)^21^. The DoG stimulus has a center white portion surrounded by darker grey areas on both sides. With a keypress, the suppressors appeared for 1 s, to induce a robust transient suppression zone in the middle part of the reference. Subjects adjusted the width between the suppressors to determine the maximum separation that still produced suppression, delimiting the borders of the suppression zone width^24^. We measured the zone in an extensive series of experiments, varying spatial frequency (0.888–11.54 cpd), contrast (12.5–100%), dominant vs. non-dominant eye, stimulus orientation (width vs height), contrast polarity (different combinations of the reference and suppressors with positive and negative contrast polarity), colored filters (matched vs. dichoptic), and ND filters (1 and 2 log units). A total of eleven normally-sighted subjects volunteered for the different sub-experiments.

## Results

### Experiment 1

#### (A) Effect of spatial frequency (n = 8)

**Figure 2:**
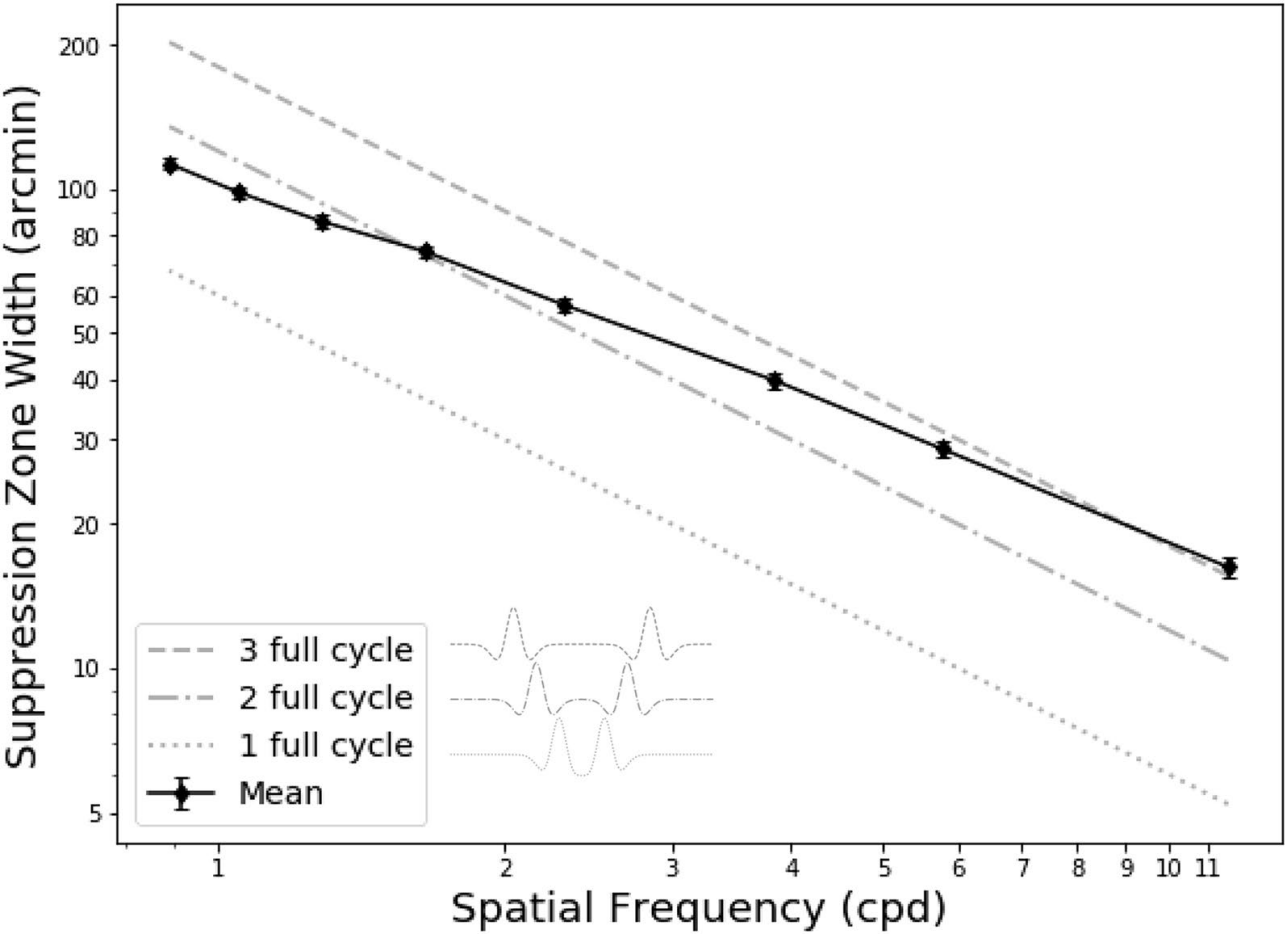
Suppression zone width as a function of spatial frequency. The solid black line is the mean values across all eight subjects. The other lines represent the size of the single, double, or triple full cycle of the DoG stimuli. The mean values are almost two full cycles of the DoG at low spatial frequencies but three full cycles at higher frequencies. The vertical error bar represents the standard error. The lower left panel illustrates the separation between the center to center of the two DoG at one, two, or three full cycles.

We found that higher spatial frequency stimuli yielded a smaller suppression zone width. Figure 2 shows the mean across all subjects with congruent white center stimuli at 100% contrast. As spatial frequency increases, the zone decreases significantly (p < 0.001). When the spatial frequency increased by 1 log unit, the zone decreased by approximately 0.83 log unit (steepness of line) when fitted with linear regression OLS on log–log coordinates. The gray lines in Fig. 2 show the corresponding size of one, two, and three full cycles of the DoG. The size of a full cycle is calculated from the spatial frequency of the DoG. For example, a DoG of 0.888 cpd will have a center width of 29.64 arcminutes and spans an area of 67.57 arc minutes for a full cycle. We found that the mean suppression zone width across all subjects was close to 2 cycles of the DoG for low spatial frequencies but closer to 3 cycles at high spatial frequencies, indicating that the suppression extent is not directly proportional to its stimulus size (i.e., not scale invariant). This differs from the findings of Liu and Schor (1994), who reported the suppression zone extent to be consistently 3 cycles of the corresponding spatial frequency. This discrepancy will be further investigated in the Discussion.

#### (B) Effect of contrast (n = 8)

**Figure 3:**
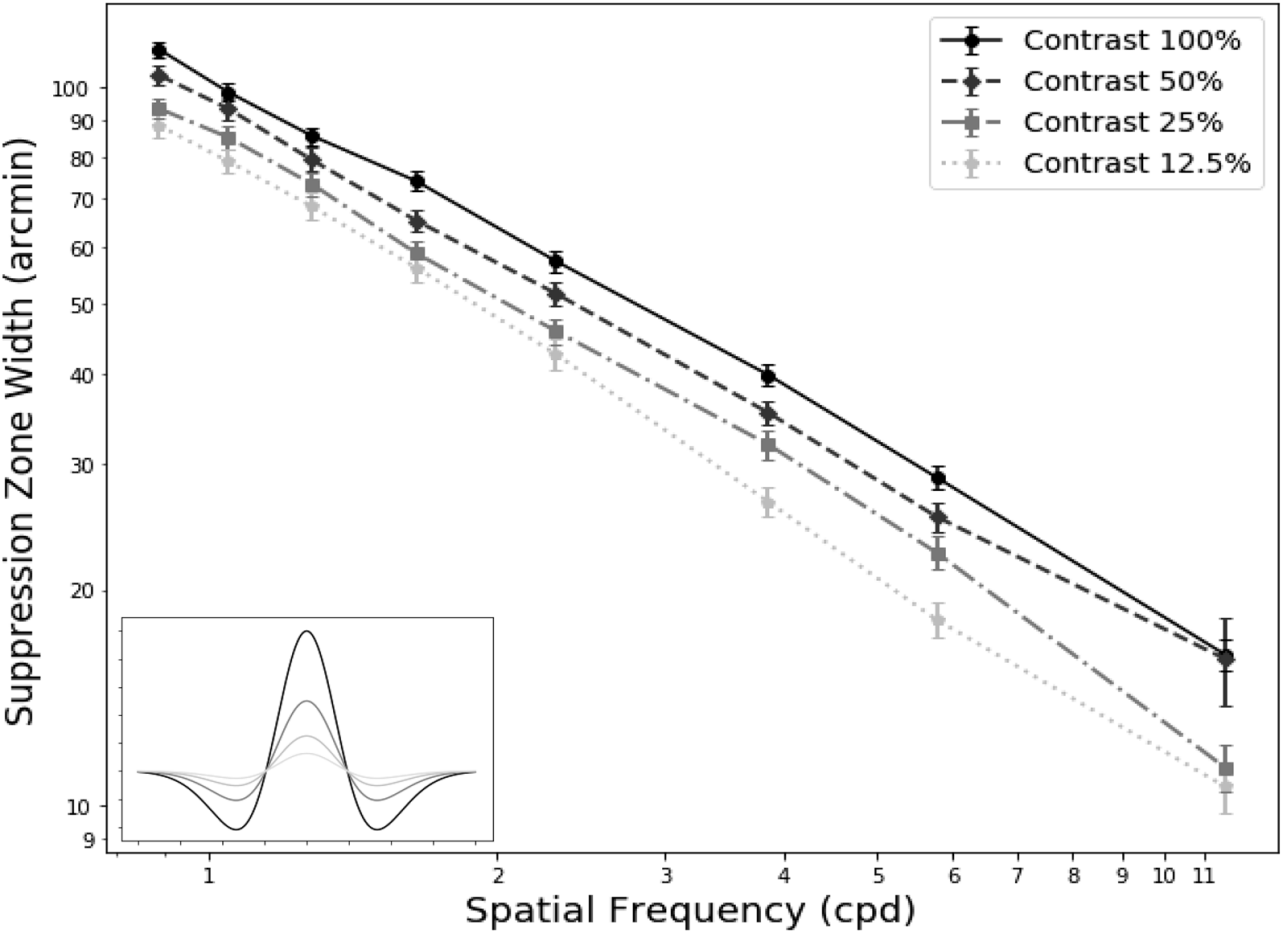
The reduction of suppression zone width as a function of contrast (n = 8). The solid black line (contrast 100%) is similar to the results obtained from Experiment 1A. This line is shifted downward with lower contrast. The vertical error bars represent the standard error. The lower left inset illustrates the cross-section view of the DoG at different contrast levels.

From Experiment 1A, we found that the zone width reduced as spatial frequency increased. With lower contrast levels (congruent in the reference and suppressors), the mean also decreased significantly (p < 0.001). Figure 3 plots the mean result across all subjects at different contrast levels. From the OLS fit, we found that for a log unit reduction of contrast, the zone decreased by 0.168 log unit in minutes. The reduction follows a similar pattern across all spatial frequencies, as shown by the downward vertical shift. All of the subjects except S07 had a similar downward shift of the slope when the stimuli were presented at lower contrast.

#### (C) Effect of eye dominance (n = 8)

When we presented the suppressors to either of the eyes, the suppression zone width did not significantly differ based on the suppressor eye when averaged across subjects (p = 0.81). This suggests that the size is similar in normal subjects irrespective of eye dominance. We also analyzed the effect of ocular dominance at different contrasts and confirmed that the zone is not affected by ocular dominance. The spread of the suppression zone width was independent of eye dominance across all contrast levels and spatial frequencies.

We fitted the results in Experiment 1 with an Ordinary Least Square (OLS) linear model to predict the suppression zone width based on the different parameters: spatial frequency, contrast, and eye dominance. The model was well fitted with a significant regression equation F (3, 2796) = 2929 (p < 0.001) with an R^2^ value of 0.759 (Adj. R^2^ = 0.758). The predicted log10 (Zone width, in arcminutes) is equal to 2.024–0.830 * log10 (Spatial Frequency, in cycles per degree) + 0.1678 * log10 (Contrast, Weber proportion) + 0.0016 (Dominant Eye). Out of the three parameters, only spatial frequency and contrast were significant predictors of the spatial extent of suppression.

### Experiment 2

#### (A) Effect of orientation (n = 8)

**Figure 4:**
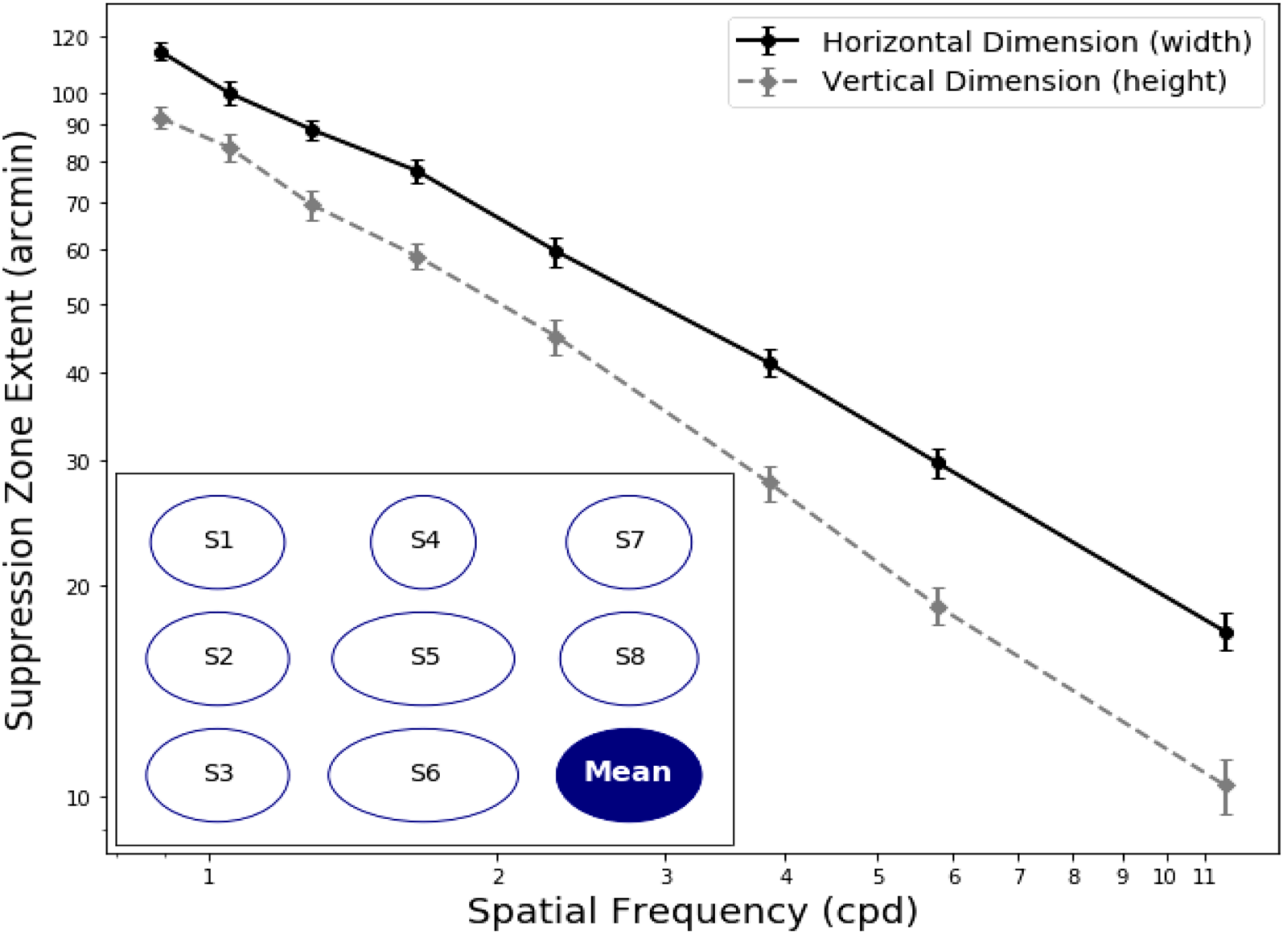
This plot shows the width and height of the suppression zones across all the spatial frequencies. The width was larger by approximately 1.4 × than the height, suggesting that the suppression zone is elliptical in shape. The schematic on the lower left shows the ratio of width to height for each subject. The mean area of suppression is elliptical in shape (dark blue). Standard errors are shown on the vertical bars.

To investigate the effect of different orientation and spatial frequency, we fitted the result with a similar OLS model. This model was well-fitted with an equation F (2, 877) = 1654 (p < 0.001) with an R^2^ value of 0.790 (Adj. R^2^ = 0.790). The intercept coefficient for the vertical dimension (height) was − 0.1384 when compared to the horizontal dimension (width). We found an asymmetric pattern in the suppression zone: the width was larger than the height. This effect manifested across most of the subjects (n = 8) and is shown in Fig. 4. Out of the eight observers, 7 observers had a horizontal to the vertical ratio in the range of 1.21–1.84. The mean ratio across all subjects at all spatial frequencies was 1.41 ± 0.26 (standard deviation). In other words, the suppression zone width was about 1.4 times larger than its height. Therefore, the area of suppression is elliptical in shape and not circular. Only subject S4 had a slightly circular suppression zone. The individual data for mean ratio of the width to height is listed in the last column of Table 1.

**Table 1:** Clinical data for each subject.

Subject ID	Age (year)	Visual acuity	Stereo Acuity (arc seconds)	Ocular Dominance @ 1 m	Experiment	Mean ratio of suppression zone (width/height)
S1	30	OD: 20/20 OS: 20/20	40	Left	1, 2 and 3	1.30
S2	25	OD: 20/16 OS: 20/17	30	Right	1, 2 and 3 except condition 2B	1.39
S3	46	OD: 20/16 OS: 20/24	40	Right	1, 2	1.38
S4	24	OD: 20/18 OS: 20/17	30	Left	1 and 2 except condition 2B	1.02
S5	58	OD: 20/18 OS: 20/15	20	Left	1, 2	1.77
S6	30	OD: 20/21 OS: 20/31	40	Right	1, 2	1.85
S7	23	OD: 20/19 OS: 20/12	30	Right	1, 2 and 3	1.22
S8	24	OD: 20/14 OS: 20/57	30	Right	1, 2	1.34
S9	34	OD: 20/50 OS:20/20	32	Left	3	–
S10	32	OD: 20/20 OS:20/20	25	Left	3	–
S11	25	OD: 20/20 OS:20/20	40	Right	3	–

The mean ratio of suppression zone width to its height for each subject in Experiment 2 is listed in the last column.

#### (B) Effect of contrast polarity (n = 6)

We also tested the stimuli at 100% contrast with four different combinations of contrast polarity: White-White, Black-Black, Black-White, and White-Black. We fitted the data in another OLS model because the original model only includes White-White stimuli. We included 2 independent parameters in this model: spatial frequency and contrast polarity (4 categorical). The model was well-fitted with an equation F (4, 1275) = 836.2 (p < 0.001) with an R^2^ value of 0.724 (Adj. R^2^ = 0.723). In relation to Black-Black stimuli, the difference in intercept coefficients were 0.0096, 0.0538, and 0.0513 for Black-White, White-Black, and White-White, respectively. Thus, the two negative contrast polarity stimuli were significantly different from the two positive contrast polarity, White-White (p < 0.001) and White-Black (p = 0.001). With a black center reference, the width was slightly smaller than the white center reference. For illustration, the stimuli in Fig. 1c,d yielded a smaller suppression zone width than the stimuli in Fig. 1a,e. This result suggests that the contrast polarity of the reference may determine the spread of suppression.

### Experiment 3

#### (A) Effect of colored filters (n = 6)

To investigate the effect of colored filters used in clinical tests, we repeated the experiments with colored anaglyph. Colored filters reduced the luminance by 1 log unit, therefore we included 1ND filter for comparison. From the result, we fitted a mixed linear regression model with 6 categorical levels: Baseline (no filter), binocular 1ND, binocular 2ND, matched red in both eyes, matched green in both eyes, and dichoptic red-green filters (red in one eye, green in the other). Compared to the isoluminant 1ND filter, the zone was not statistically significant with matched red filter (p = 0.712), matched green filter (p = 0.503), and dichoptic red-green filter (p = 0.250), suggesting that colors do not affect the suppression zone in a consistent manner. When we compared matched (only red or green) to dichoptic colored filters, the results were near significance (p = 0.063). Four out of the six subjects had individual p < 0.023 suggesting individual variability (three subjects had larger suppression size while one had a smaller size).

#### (B) Effect of luminance (n = 6)

From the model, we do not find any significant difference in the width as a function of luminance (p > 0.05). When either 1 or 2ND filters were placed in front of the two eyes, the suppression zone width remained robust and is independent of the luminance level despite a reduction of 2 log units.

## Discussion

In a series of experiments, we determined that the spatial extent of suppression depends on the properties of the stimuli used to instigate rivalrous suppression. Of all the factors, the spatial frequency of the stimulus affects the width of suppression the most. Our finding agrees with other studies, in which finer stimuli had a smaller suppression area compared to coarse stimuli^21,25^. Even though the size of suppression varies from subject to subject, the changes as a function of spatial frequency were consistent across all subjects. Although we used the same set of spatial frequencies as Liu & Schor (1994), our results for the overall suppression zone width were smaller, especially at lower spatial frequencies. The different response methods used may be the reason behind this discrepancy. They used a forced-choice Method of Limits while we used a Method of Adjustment which has been reported to yield smaller estimates because of the nature of the underlying decision process^26^.

If suppression were based solely on stimulus characteristics, parsimony would dictate that the suppression zone should be directly proportional to the stimulus size (scale invariant)—in fact, this is what Liu and Schor found: a suppression zone of exactly three cycles of the DoG across all spatial frequencies. Instead, we found that the suppression zone width was not strictly proportional to the stimuli, having a smaller proportion at lower spatial frequencies (Fig. 2). The solid line in Fig. 2 shows that the suppression zone width deviates from this scale-invariance, since it is not parallel with any of the broken lines, which have a slope of − 1. This lack of scale-invariance is similar to that reported previously by Georgeson and Wallis (2014)^27^, who asked subjects to report their perception of Gaussian-blurred edges presented dichoptically, which could appear to be fused, doubled, or offset and suppressed. Although the experimental conditions and response paradigm differed from our study, their inferred suppression widths also deviated from scale invariance, although with a flatter slope (absolute value 0.33) than we found (absolute value 0.83); see their Fig. 8.

**Figure 5:**
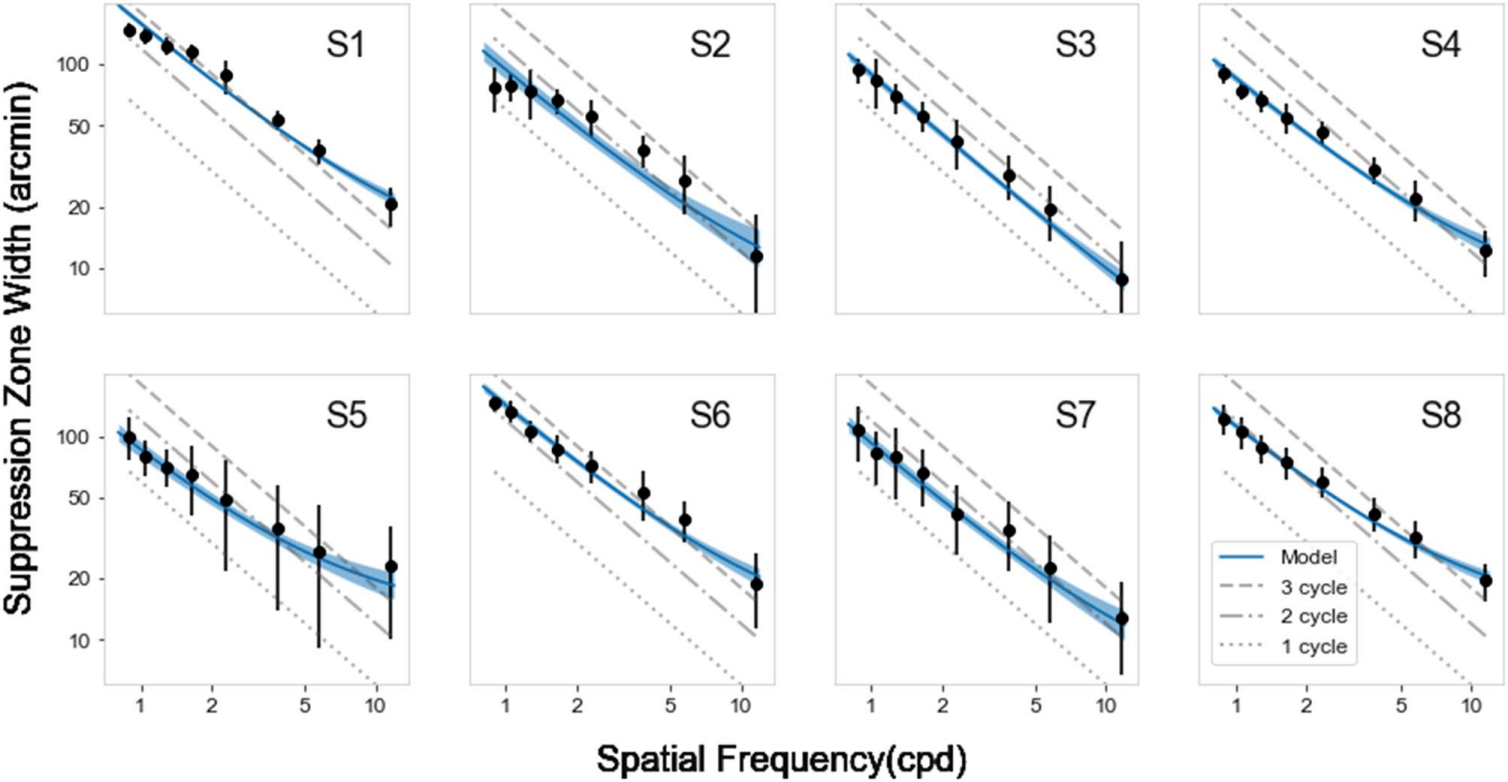
The model predicts the individual results well, indicated by the shaded blue region, indicating 95% credible intervals from the fits. Different dashed lines indicate the multiplication of the number of full cycles.

**Figure 6:**
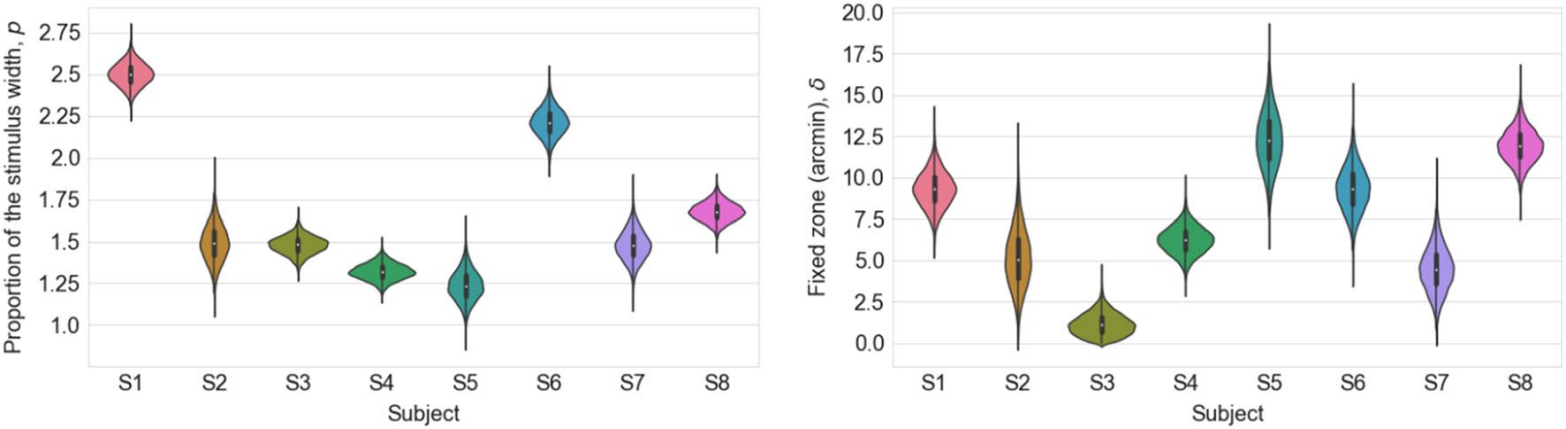
In the model, the parameter estimates for the proportion of stimulus width, *p* is between 1.25 and 2.5 cycles (left panel). The constant term delta is 5–12.5 arc minutes for most subjects (right panel).

To further investigate the departure from scale-invariance, we fit each subject’s data with a log-transformed linear model of the form log (Suppression Zone Width) = log ( p*(B*2.28) + delta ), where p is a subject-dependent proportion of cycles of the DoG spatial frequency (B = central lobe width and B*2.28 is one cycle, as specified in Eqs. 1–4), and delta is a subject-dependent offset that is common across spatial frequencies. The “p" term can be understood as the suppression zone width relative to the size of the stimulus. We fit this model using PyMC3^28^, which uses Markov chain Monte Carlo to determine optimal parameters for generic mathematical models with minimal assumptions. Although PyMC3 incorporates Bayesian principles, we used flat (uninformative) priors. The model fit the individual data well, as shown by the 95% confidence intervals in Fig. 5 (shaded blue regions), resulting from chains of length 2000. Figure 6 shows the two model parameters for each subject: the individual's baseline proportion of the stimulus size and the common fixed zone. The stimulus-dependent component, p, is 1.5–2.5 (cycles). The model’s constant term ranges between 5 and 12.5 arc minutes for all subjects except S3.

**Figure 7:**
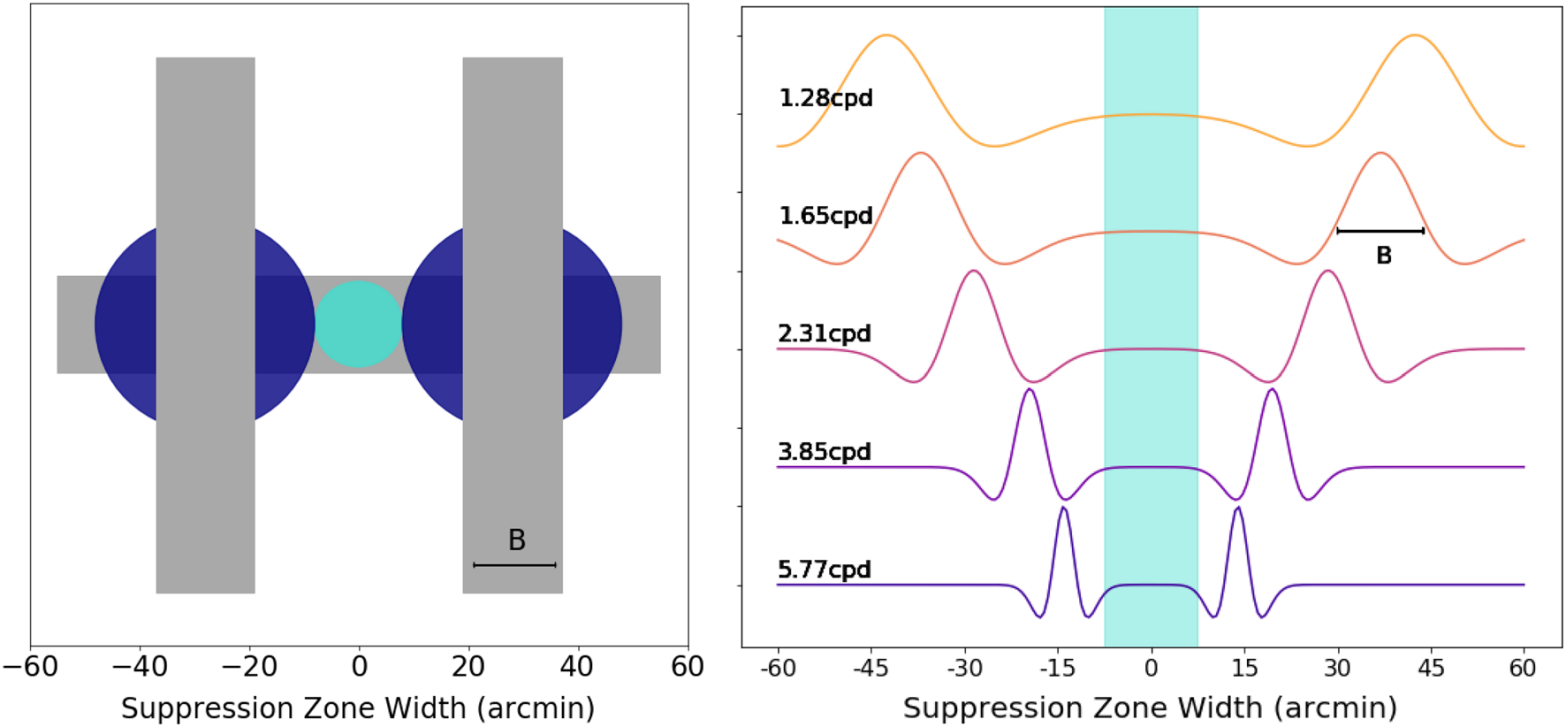
(Left panel) Illustration of the postulated suppression zone. The two vertical bars represent the suppressors while the horizontal bar represents the reference. Surrounding the suppressor, the area in dark blue represents the area of suppression which is stimulus-dependent, p. In between the dark blue areas, the cyan patch corresponds to the non-stimulus dependent component, delta (edge-to-edge separation, ES), where subjects were asked to fixate during the experiment. (Right panel) Illustration of the edge-to-edge separation at different spatial frequencies. The ES is scale invariant, as indicated in the shaded cyan region. We only include a few spatial frequencies for illustration purposes. B is the width of the center part of the DoG.

For example, for subject S1, the suppression zone was 2.5 cycles of the DoG, with a fixed zone of ~ 10 arc minutes, meaning their zone was ~ 2.5 cycles at low spatial frequencies and more than 3 cycles at the highest spatial frequencies. Interestingly, one of Liu and Schor’s three subjects did show a pattern like this. This fixed zone can also be understood as the gap between the two suppressors, when the innermost edges of the DoGs are lined up to equivalent points on their envelope at each spatial frequency, as illustrated in Fig. 7 (right panel). This suggests the contribution of two elements to the spatial extent of suppression, as shown schematically in Fig. 7 (left panel): a stimulus-dependent component that scales with the stimulus size that spreads from the locus of dissimilarity (dark blue circles, emanating from the intersection point of suppressors and reference, which is *p*) and a non-stimulus-dependent component (cyan region, centered at fixation, *delta*). The fixed zone, which explains the deviation from scale invariance and relatively broader width at high spatial frequencies, may be related to fixation disparity at the fovea. Coincidentally, with very fine stimuli (higher spatial frequency), Kaufman also reported that the strongest suppression occurred when the gap between the suppressors was 14 arc minutes^19^, close to the value of our non-stimulus dependent zone (delta).

We found that the extent of suppression decreased when the contrast of the reference and suppressor were reduced congruently. Similarly, low contrast orthogonal gratings have been found to fuse more easily into plaid patterns, whereas those with higher contrast tend to be rivalrous^29,30^. Our data agree with the general finding that rivalry is less pronounced at lower contrast. Since rivalry is more pronounced with higher contrast, it has been commonly associated with the parvocellular pathway^31,32^. However, stimuli with higher spatial frequencies are fused more easily than lower spatial frequencies, implying the involvement of the magnocellular pathway^33^. In our study, the suppression zone width was largest at highest contrast (implying parvocellular) and lowest spatial frequency (implying magnocellular), in agreement with another study that suggested that rivalry is not strictly limited to either of the pathways^34^. As for contrast polarity, we found that the suppression area is slightly smaller with negative polarity stimuli than with positive contrast polarity stimuli. One possible explanation is a difference in the suppression mechanism between the ON versus the OFF pathway, for example due to differences in receptive field sizes^35^.

**Figure 8:**
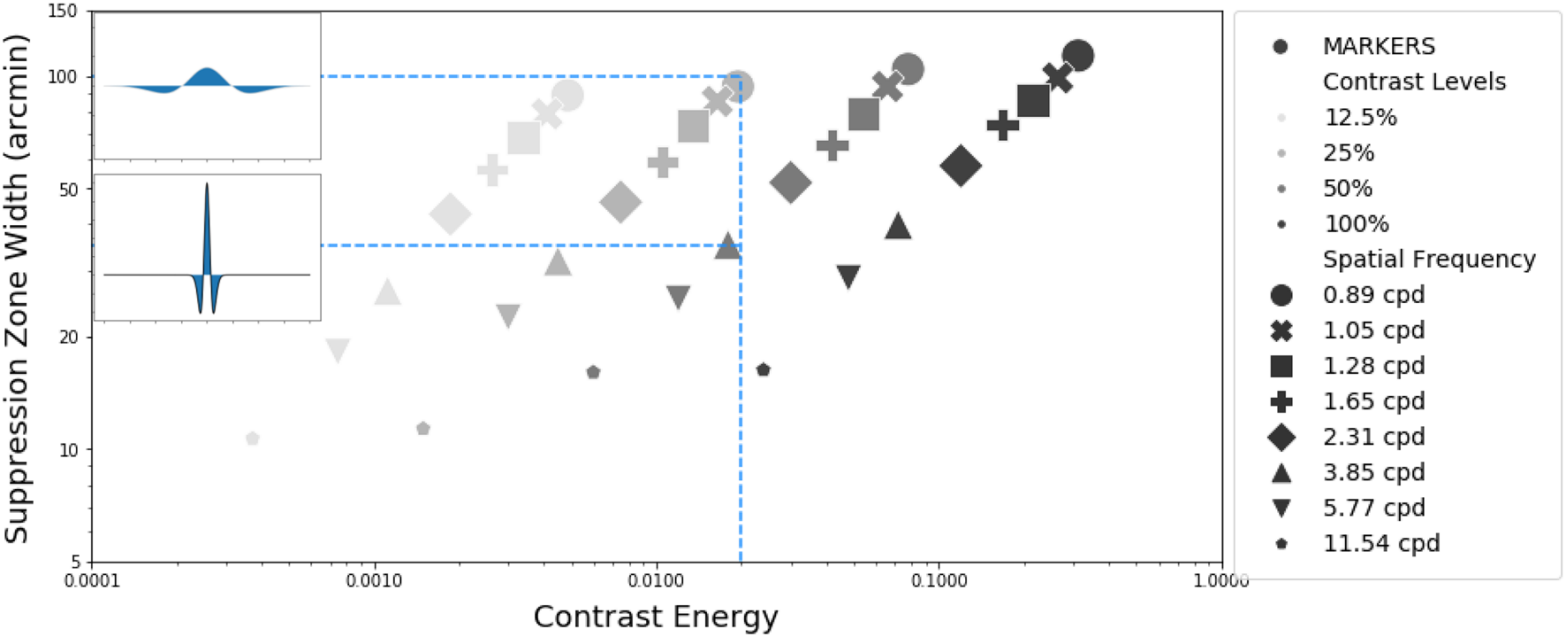
This plot illustrates how suppression zones change as a function of contrast energy. Different shapes of the markers indicate different spatial frequencies while the grayscale shades indicate the contrast level. Any contrast energy level could result in multiple suppression zones. The inset shows that a DoG at lower spatial frequency with low contrast can have equivalent contrast energy to a DoG at a higher spatial frequency with high contrast. The suppression zone width can vary even with a single value of contrast energy. Contrast energy alone is not enough to explain the spatial extent of the suppression zone.

Note that in our stimulus, when the spatial frequency changed, so did the stimulus size. For example, higher spatial frequency DoGs are narrower than low spatial frequency DoGs, and thus the total contrast energy (defined as the integral of the squared local contrast over the stimulus envelope) is also lesser. The measure of contrast that captures the dependence on stimulus size is called “contrast energy”. The inset to Fig. 8 shows that two stimuli (low spatial frequency with low contrast vs. high spatial frequency with high contrast) could have equivalent contrast energy. Could differences in contrast energy explain our results? The central panel in Fig. 8 replots the results from Fig. 3 with contrast energy (instead of spatial frequency) on the x-axis. For example, a contrast energy value of 0.02 on the x-axis (vertical line in Fig. 8) could result from different combinations of spatial frequency and contrast levels. These combinations could have different suppression zone widths (two horizontal lines in Fig. 8), revealing a lack of a simple one-to-one correlation between contrast energy and suppression zone width. Thus, contrast energy alone cannot account for the changes in the suppression zone width in our experiment. Future work with modified stimuli is needed to decouple the effects of contrast, spatial frequency, size, and physical contrast.

Many aspects of binocular vision and vergence are more extensive in the horizontal meridian. For example, Panum’s fusional area and range of fusional vergence is larger in the horizontal than the vertical dimension^36^. Similarly, we found that the suppression area is not circular but elliptical in shape, with a horizontal dimension larger than vertical across all spatial frequencies, in agreement with the result found by Kaufman (1963)^24^. Another study which used similar orthogonal stimuli found that normalized suppression time is also longer in the horizontal dimension than vertical^37^. In contrast to Liu & Schor, we did not find that the suppression zone was vertically elongated at low spatial frequencies and horizontally elongated at high spatial frequencies.

What could have contributed to the anisotropy of the suppression zone? Kaufman proposed vergence error, while Liu & Schor argued that if there is vergence error, the suppression area would be a constant value corresponding to the amount of individual vergence error. Vergence error would also lead to double vision. To avoid this, our stimulus had an outer binocular fusion lock and two vertical fixation lines pointing towards the middle region of the reference. None of our subjects reported double vision or difficulty fusing the stimuli. Additionally, our setup allows a more natural vergence and accommodation than a haploscope system. While the effect of vergence should be minimal due to the use of a long horizontal reference bar, to precisely study the relationship between vergence control and the spread of suppression as proposed by Kaufman, recording the binocular eye position is needed. In addition to the vergence system, there are a multitude of horizontal versus vertical anisotropies in the visual pathway, including cone density and ganglion cell distribution^38,39^ that may contribute to the elliptical suppression zone.

Any additional spatial features close to the suppression area may affect the outcome^37^. With that in mind, we set the width of the reference to be equal to the height of the suppressors while in Liu & Schor (1994), the reference was longer than the suppressors. To investigate if the sharp edges of the suppressors would increase saliency and affect the outcome or explain the difference between their result and ours, we conducted a control experiment with different vertical suppressor heights (from 1.67° to 16.7°) while the length of reference was fixed at 8.5°. Three subjects (S1, S2, & S4) participated in this experiment and we found that the size of the suppression zone was independent of the suppressor length (Repeated measure ANOVA, F (9,18) = 0.60, p = 0.78).

With colored filters, the area of suppression is similar to isoluminant achromatic stimuli. With dichoptic chromatic red-green filters, we did not find any significant difference. However, when analyzed individually, we found individual variability. This implied that the effect of color rivalry is not universal across subjects, but likely affected by individual differences, including fusional vergence. It is worth noting that these subjects have normal binocular vision (normal visual acuity and stereopsis, and no strabismus). In the case of individuals with intermittent exotropia with weak fusional vergence, color rivalry could inadvertently dissociate binocularity and facilitate suppression. Color difference induces stronger rivalry and reduces fusion because of inhibitory mechanisms from chromatic-sensitive neurons in the visual cortex^40,41^. Besides the colored filters, we also found that the suppression zone width is independent of luminance and eye dominance. Considering that lower luminance delays visual signals^42^, we were surprised that the suppression zone width was not affected. The overall luminance reduced with ND filters, but the contrast ratio between the stimuli and background remained constant.

There is a mixed literature debating if the suppression during binocular rivalry has the same mechanism as amblyopia since amblyopes have limited binocular function^12,43,44^. Since inhibition is one of the proposed mechanisms underlying the deficiencies in amblyopia^45^, our view is that interocular inhibition may actually originate from binocular rivalry at the very early stage. In individuals with good binocular vision, both eyes have roughly equal reciprocal strength of inhibition. In the case of amblyopia, the amblyopic eye may have a weaker inhibitory strength compared to the non-amblyopic eye^46–48^. However, under balanced conditions between the two eyes, such as contrast balancing or penalizing the fellow eye with a lower contrast level, amblyopes also experience alternating suppression, similar to binocular rivalry of normal observers^49,50^. These two types of suppression have shown similar traits: spatial-frequency^51,52^ and scale-dependence^53^. Transient suppression or binocular rivalry is thought to reflect competition between monocular neurons within the primary visual cortex^54^. It is quite possible that constant suppression arises from transient suppression, but deepens with time and spreads into higher cortical areas.

In summary, we found that the spatial extent of suppression is not fixed, but changes with stimulus parameters (spatial frequency, contrast, and contrast polarity). It is independent of eye dominance, luminance and colored filters. Therefore, the stimulus used to assess suppression is crucial to its outcome. Unlike a visual field scotoma which has a fixed size, a suppression scotoma changes based on what is being seen. These findings set a lower limit on the generality of suppression size measurement, and may help to understand the discrepancy found between the different clinical and laboratory-based tests.

## Methods

### Participants

We recruited a total of 11 subjects with normal binocular vision and typical stereopsis (no eye diseases, amblyopia or strabismus). All had good distance visual acuity (at least 20/32) except for two subjects (S8 & S9) whose glasses were not fully corrected for distance but had good near acuity (20/20), which is sufficient for the experimental set-up (~ 1meter). Their visual acuity can be improved to 20/20 with refraction. Subjects were either emmetropes or wore their habitual correction during the experiment. Eight of the subjects were male, and three were female (mean age = 31.9 ± 10.91 year old). Subject S5, a presbyope wore additional plus lenses corrected for 1 m. Table 1 shows the data for all the subjects. Three of the subjects (S1, S3 & S5) are the authors of this paper, while the other subjects were naïve observers. These data were collected from two different sets of experiments; Experiment 1 and 2 were done together, while Experiment 3 was done later with additional subjects. Some of the subjects from Experiment 1 and 2 participated in Experiment 3. Two subjects (S2 and S4) were assigned to participate in a control experiment (see “Discussion”) rather than Experiment 2B. Written informed consent was obtained from all participants before the experiment, and the experimental procedures were approved by the Institutional Review Board of the University of Houston. All experimental procedures were performed in accordance with this protocol.

### Preliminary assessment

We measured monocular visual acuity using the computer-based FrACT 3.10.5 acuity test (Acuity Landolt C with 4 directions)^55^ and stereo acuity with the Wirt Circle in Stereo Fly Test. Motor eye dominance was performed using the hole-in-card method to determine ocular dominance. We tested eye dominance at 6 m and also 1 m, which is the distance from the display screen to the observer during the experiment. For all the subjects, the dominant eye was the same for both distances. Proper demonstrations and explanations were given to ensure participants understood the task.

### Set-up

We used a PROPixx DLP LED Projector (VPixx Technologies Inc) to rear-project the stimuli on a large projection screen. Subjects sat 1.03 m away from the screen. Each pixel on the screen subtended one arc minute. The screen resolution was 1920 × 1080 pixels and subtended a total angle of 32° × 18°. The projector has a linear contrast response (gamma), confirmed with a Konica Minolta LS-160 photometer. A circular polarizer, which temporally switched between the left and right eye images at 120 Hz, was used to present the stimuli dichoptically. Subjects used a chinrest and performed the task with passive 3D glasses along with their optical correction. The luminance on the projector screen was approximately 320 cd/m^2^ and reduced to 145 cd/m^2^ with the polarized glasses. During the experiment, the room was completely dark except for the projector.

### Stimulus

To provide robust interocular suppression while allowing parametric modulation of low-level stimulus parameters we adapted the stimuli used by Liu & Schor (1994)^21^. The original stimulus is from Kaufman (1963) where rivalrous images were presented to either eye^24^. One eye will see two vertical lines while the other sees a horizontal line. Because of the disparate images, at the intersection between the lines, an area of suppression occurs. When all lines are presented simultaneously, the suppression scotoma alternates between the two eyes. To manipulate which stimuli are suppressed, the horizontal line (“reference”) can be kept constant, such that the temporal onset of the vertical lines (“suppressors”) will induce suppression of the horizontal line.

The stimuli were drawn with Psychopy software^56^ and based on Difference of Gaussians.

1 $$DoG\left( x \right) = 3^{{\left( {\frac{{ - x^{2} }}{{\sigma^{2} }}} \right)}} - 2^{{\left( {\frac{{ - x^{2} }}{{2.25\sigma^{2} }}} \right)}}$$

2 $$\sigma = \frac{B}{1.75}$$

The DoG appeared as a white center bar flanked by darker bars on each side. The stimuli have a bandwidth of 1.75 octaves. B is the width of the center peak of the DoG to be drawn in pixels or arc minutes:

3 $$B\left( {degree} \right) = Width\left( {min} \right) \times \frac{2.28}{{60}}$$

The dominant spatial frequency of each stimulus is calculated based on this formula:

4 $$Spatial Frequency\left( {cpd} \right) = \frac{1}{{\left( {2.28 \times B} \right)}}$$

As shown in Fig. 1, the single horizontal bar (width = 8.5°) served as a reference bar. It was continuously visible while two vertical bars (height = 8.5°) served as suppressors, appearing symmetrically to the left and right of the center of the screen for a duration of 1 s. Two vertical fixation lines were drawn to indicate the fixation area and participants were instructed to keep their fixation between these two lines during the experiment. An outer square (15° × 15°) served as a binocular fusion lock. Each time the subject pressed a key, the onset of the suppressors induced robust transient interocular suppression at the intersection with the reference bar. The task was to align the suppressors by turning a knob (Method of Adjustment) so that the middle part of the reference bar just barely disappeared. The position of the suppressors was randomly assigned at the beginning of each trial with a maximum of 3.3-degree separation. This transient suppression usually lasted less than a second. During the suppression period, each suppressor induced a circular zone of suppression at the intersection and the inner part of the reference bar was suppressed and disappeared (as outlined by the yellow dotted line in Fig. 1b). The distance between the center of the two suppressors is termed as the suppression zone width, in arc minutes. This distance between the suppressors is in fact the diameter of the suppression zone: the sum of a radius from each of the two suppressors.

To standardize the criterion across the subjects, they were instructed to adjust the position of the suppressors inward from the outermost position. After three consecutive onsets to ensure robust suppression, subjects pressed another key to save the distance between the suppressors and advance to the next condition. To control for contrast adaptation to the horizontal reference bar, after each trial, a grey background appeared. Subjects were free to move their eyes freely between trials but instructed to fixate at the fixation area when the suppressors appeared.

### Experimental conditions

Experiment 1 and Experiment 2 were performed together with a total of 3 blocks (all the conditions in Experiment 1 were grouped together as a block, and two blocks of different conditions in Experiment 2) while Experiment 3 (six blocks for different filters) was performed later with additional subjects. The order of the blocks and conditions was randomized. Each block was repeated five times, yielding five measurements for each condition.

#### Experiment 1

Experiments 1A, B, and C were grouped and performed together as a block (8 spatial frequencies × 4 contrast levels × 2 eyes = 64 trials per block). Each trial can vary by spatial frequency, contrast level or viewing eye. Both the reference and suppressor bars have similar contrast and a white center contrast polarity.

A. *Effect of spatial frequency *We varied the spatial frequencies of the stimuli congruently (both suppressors and reference were always presented with similar spatial frequency). The spatial frequencies tested were 0.888, 1.049, 1.282, 1.649, 2.308, 3.847, 5.77 and 11.54 cpd.
B. *Effect of contrast* Four different congruent stimulus contrast levels were used (100%, 50%, 25%, and 12.5% Weber contrast) to investigate the effect of contrast for all the spatial frequencies tested in Experiment 1A. The contrast is defined as [(peak luminance of the center DoG—luminance of grey background)/ mean luminance].
C. *Effect of eye dominance* The vertical suppressors were randomized between the two eyes (dominant and non-dominant) to study the effect of eye dominance.

#### Experiment 2

A. *Effect of orientation (width vs height)* We rotated the stimuli 90° to study the vertical dimension of suppression. The constant reference was oriented along the vertical meridian while the double suppressors appeared parallel to the horizontal meridian. The height of the area of suppression was the vertical space between the double horizontal suppressors. Each block consists of 8 spatial frequencies at 100% contrast and a white center. In the analysis, we compared the height of the suppression zone to its width drawn from Experiment 1A data.
B. *Effect of contrast polarity* We tested four different combinations of contrast polarity: white-white (both reference and suppressors have a white center), black-black (both have a black center), white-black (reference with white center while suppressors have a black center) and black-white stimuli (reference has black center while suppressors have a white center). We tested the different combinations with all eight spatial frequencies at 100% contrast (8 spatial frequencies × 4 contrast polarity = 32 trials per block).

#### Experiment 3

A. *Effect of color filters* We tested the same stimuli with colored filters at 0.888, 2.308, and 5.77 cpd (100% contrast, white center bars). Subjects wore the colored filters on top of the polarized glasses. In the matched chromatic filter condition, the color of the filters (red or green) were the same in both eyes while in dichoptic chromatic filters (commonly used for anaglyph), the red filter was placed in front of the right eye while the green filter was placed in front of the left eye. The colored filters were cardboard consumer anaglyph filters. By adding the colored filters, the average luminance reduced from 145 to 15 cd/m^2^ (approximately 1 log unit reduction) for both colors. To isolate the effect of chromaticity, the result with matched chromatic red or green filters were compared to a 1ND filter (15 cd/m^2^) in Experiment 3B. The subjects adapted to the filters for a minimum of three minutes to adjust for the luminance before each experiment, based on typical cone pigment regeneration duration (3–5 min).
B. *Effect of luminance* We repeated Experiment 3A (same contrast level and spatial frequencies) with 1ND (15 cd/m^2^) and 2ND (1.5 cd/m^2^) filters. The ND filters were placed on a holder and positioned in front of the subjects. Similarly, subjects adapted for a minimum of three minutes prior to the experiment.

### Data analysis

We performed data analyses using Python, the NumPy/Scipy scientific libraries, and Statsmodels^57^. To identify which conditions contributed to the suppression zone width, we fit the results of Experiment 1, 2, and 3 with multiple linear regression using ordinary least squares (OLS).
